# Supplementing Glycine and N-acetylcysteine (GlyNAC) in Aging HIV Patients Improves Oxidative Stress, Mitochondrial Dysfunction, Inflammation, Endothelial Dysfunction, Insulin Resistance, Genotoxicity, Strength, and Cognition: Results of an Open-Label Clinical Trial

**DOI:** 10.3390/biomedicines8100390

**Published:** 2020-09-30

**Authors:** Premranjan Kumar, Chun Liu, James W. Suliburk, Charles G. Minard, Raja Muthupillai, Shaji Chacko, Jean W. Hsu, Farook Jahoor, Rajagopal V. Sekhar

**Affiliations:** 1 Translational Metabolism Unit, Division of Endocrinology, Diabetes and Metabolism, Department of Medicine, Baylor College of Medicine, One Baylor Plaza, Houston, TX 77030, USA; premranjan.kumar@bcm.edu (P.K.); chunl@bcm.edu (C.L.); 2 Department of Surgery, Baylor College of Medicine, One Baylor Plaza, Houston, TX 77030, USA; Suliburk@bcm.edu; 3 Institute of Clinical and Translational Research, Baylor College of Medicine, One Baylor Plaza, Houston, TX 77030, USA; minard@bcm.edu; 4 Baylor-St. Luke’s Medical Center, Houston, TX 77030, USA; rmuthupillai@uh.edu; 5 USDA/ARS Children’s Nutrition Research Center, Baylor College of Medicine, Houston, TX 77030, USA; schacko@bcm.edu (S.C.); jeanweih@bcm.edu (J.W.H.); fjahoor@bcm.edu (F.J.); 6 Thomas Street HIV-Health Center, Harris Health, Houston, TX 77009, USA

**Keywords:** premature aging, HIV, mitochondrial function, oxidative stress, GlyNAC, Glutathione, cognition, physical function

## Abstract

**Background:** Patients with HIV (PWH) develop geriatric comorbidities, including functional and cognitive decline at a younger age. However, contributing mechanisms are unclear and interventions are lacking. We hypothesized that deficiency of the antioxidant protein glutathione (GSH) contributes to multiple defects representing premature aging in PWH, and that these defects could be improved by supplementing the GSH precursors glycine and N-acetylcysteine (GlyNAC). **Methods:** We conducted an open label clinical trial where eight PWH and eight matched uninfected-controls were studied at baseline. PWH were studied again 12-weeks after receiving GlyNAC, and 8-weeks after stopping GlyNAC. Controls did not receive supplementation. Outcome measures included red-blood cell and muscle GSH concentrations, mitochondrial function, mitophagy and autophagy, oxidative stress, inflammation, endothelial function, genomic damage, insulin resistance, glucose production, muscle-protein breakdown rates, body composition, physical function and cognition. **Results:** PWH had significant defects in measured outcomes, which improved with GlyNAC supplementation. However, benefits receded after stopping GlyNAC. **Conclusions:** This open label trial finds that PWH have premature aging based on multiple biological and functional defects, and identifies novel mechanistic explanations for cognitive and physical decline. Nutritional supplementation with GlyNAC improves comorbidities suggestive of premature aging in PWH including functional and cognitive decline, and warrants additional investigation.

## 1. Introduction

Effective antiretroviral therapy has improved the health and life expectancy of people with HIV (PWH) [1,2]. However, accumulating evidence indicates that younger PWH in the 45–60 year age-group develop abnormalities typically associated with HIV-uninfected geriatric adults over 70 years of age, suggesting premature aging in PWH [3,4,5,6,7]. For example, a recent study in 336 young PWH (median age 44 years) found that compared to age, sex, and ethnicity-matched uninfected controls, the burden of geriatric conditions was significantly higher in PWH, together with lower quality of life scores, 5-fold higher healthcare utilization, and 4-fold higher mortality [6]. Other reports have linked early aging in PWH to diverse health conditions ranging from declining gait speed [8], physical function [9] and cognition [10,11], mitochondrial aging [12,13], and elevated inflammation [14,15,16]. Additional evidence for early aging in PWH comes from studies in corneal epithelial cells, monocyte, and immune dysfunction, and the emergence of frailty at an earlier age [6,7,17]. Although premature aging in PWH is now being recognized as a new, significant public health challenge, there is limited knowledge about underlying causal mechanisms, and effective interventions are lacking.

Clues to understand potential contributors to early aging in PWH come from the two cardinal ‘free radical’ and the ‘mitochondrial’ theories of aging, which suggest that elevated oxidative stress (OxS) [18] and mitochondrial dysfunction [19] are key factors responsible for geriatric aging. Interestingly, both elevated OxS and impaired mitochondrial fuel oxidation are present in PWH [13], which raises the important question on whether they contribute to early aging, and whether correcting these twin defects in PWH could improve or reverse premature aging. Elevated OxS is caused by excess mitochondrial reactive oxygen species and has been linked to mitochondrial damage [20]. In mitochondria, the initial defense against OxS comes from the enzyme superoxide-dismutase which detoxifies the harmful superoxide radical to hydrogen-peroxide. However, hydrogen-peroxide is still extremely toxic and requires further detoxification to water, and this is provided by a tripeptide protein called glutathione (GSH, γ-glutamyl-cysteinyl-glycine), the most abundant endogenous intracellular antioxidant protein. GSH deficiency is reported in PWH [13], but its connection to mitochondrial dysfunction is not well explored. Acute induction of cellular GSH deficiency results in apoptosis or mitochondrial injury [21,22]. In translational studies in aged mice, we discovered that inducing GSH deficiency results in impaired mitochondrial fatty-acid oxidation (MFO), and that correcting naturally occurring GSH deficiency improves defective MFO [23]. In a small pilot study in such ‘older’ PWH with premature aging, we investigated the mechanisms contributing to GSH deficiency and its link with mitochondrial fat and glucose oxidation, and reported that (a) GSH deficiency in PWH occurs due to diminished synthesis caused by deficiency of its precursor amino-acids glycine and cysteine [13]; (b) supplementing GlyNAC (combination of glycine and N-acetylcysteine, a cysteine donor) for 2 weeks improved red blood cells (RBC)-GSH synthesis, RBC-GSH concentrations, mitochondrial fuel oxidation, and muscle (grip) strength [13], and lowered inflammation [14]. Based on these observations, we hypothesized that (a) PWH in the 45–65 year age group will have GSH deficiency and multiple defects associated with premature aging; (b) these defects could be improved/reversed with a longer 12-week duration of oral, nutritional supplementation with GlyNAC to correct GSH deficiency; and (c) any accrued benefits could be lost after withdrawing GlyNAC supplementation. To test our hypothesis, we conducted an open-label clinical trial comparing PWH to age, gender and body mass index (BMI)-matched uninfected controls and report our findings here.

## 2. Methods

### 2.1. Study

This exploratory pilot open-label clinical trial was approved by the Institutional Review Board for human studies at Baylor College of Medicine, and registered with clinicaltrials.gov (NCT02348775, Approved by the Baylor IRB on 4 June 2014). All participants signed written informed consent on forms approved by the Baylor Institutional Review Board.

### 2.2. Study Participants

Eight PWH (6 men, 2 women), and 8 age, gender, and BMI-matched uninfected controls, aged 45–65 years, were studied. PWH were (a) on stable antiretroviral regimens with suppressed viral loads; (b) not hospitalized for 6 months preceding the study. Participants did not have: (a) diabetes, abnormal thyroid-function tests or untreated thyroid disease, known heart disease, liver, or renal impairment (serum creatinine >1.5 mg/dL), fasting plasma triglyceride concentrations >500 mg/dL; (b) coinfection with viral hepatitis or other non-HIV infections; (c) prior treatment with testosterone, anabolic agents, or corticosteroids in the past 6 months; (d) difficulties with physical assessments; (e) treatment with anticoagulant or antiplatelet medications. To avoid potentially confounding effects on mitochondrial function, any lipid lowering agents, or non-vitamin non-prescription nutritional supplements (including but not limited to coenzyme Q, carnitine, omega fatty-acids) were stopped from 4 weeks prior to laboratory tests until completion of the study.

### 2.3. Study Protocol

Participants were studied at the Metabolic Research Unit (MRU) of the Children’s Nutrition Research Center, Baylor College of Medicine. They underwent fasting screening lab tests (fasted plasma lipid and liver profiles, blood-urea nitrogen, creatinine, thyrotropin, free-thyroxine, cortisol, glucose, glycosylated hemoglobin, blood counts), and qualifying participants entered into the study. Then they had assessment of physical function, cognitive testing, and body composition. Participants received oral deuterated water (4 g/kg body weight) the night before their MRU visit. They came in after an overnight fast, and intravenous catheters were placed in the dorsum of both hands with one hand used for blood draws (warmed with a heating pad), and the other for tracer infusions. After basal blood sampling, they received tracer infusions of ^2^H_2_-glucose (initiated at 21.6 μmol/kg; maintained at 21.6 μmol/kg/h) for 5 h, and ^2^H_3_-methylhistidine (initiated at 0.06 μmol/kg; maintained at 0.03 μmol/kg/h) for 3 h. Blood was drawn every 15-min during the final hour of tracer infusions. Indirect calorimetry was performed in the 5th hour, and then a quadriceps muscle biopsy was performed by a surgeon using a sterile, disposable Bard Biopsy System (Bard Instruments, Tempe, AZ, USA). Blood drawn was centrifuged immediately and red blood cells (RBC) and plasma stored at −80 °C for later analyses. Urine was collected for 6 h during the visit. After study completion, participants were provided a meal and discharged home. Control participants only underwent a baseline study, and were not supplemented. PWH repeated the study protocol after completing 12 weeks of GlyNAC supplementation, and again 8 weeks after stopping GlyNAC to determine the washout effects on RBC-GSH, OxS, mitochondrial function, cognition and physical function (but tracer infusions, biopsy, or dual-energy X-ray absorptiometry (DEXA) scan were not done at the 20-week visit).

### 2.4. Supplements and Monitoring

GlyNAC was provided as capsules of glycine (1.31 mmol/kg/d) and cysteine (0.83 mmol/kg/d, provided as N-acetylcysteine, NAC), prepared by a licensed compounding pharmacist, and replenished every 4 weeks for the 12-week duration of supplementation. Compliance to GlyNAC capsules was assessed with phone calls, and by counting the remaining capsules at the end of each 4-week period. Renal (creatinine) and liver function (as alanine and aspartate transaminase, ALT and AST) were monitored every 4 weeks while on supplementation.

### 2.5. Outcome Measures

#### 2.5.1. Glutathione Concentrations and Oxidative Stress

Muscle GSH concentrations, and RBC total and reduced glutathione concentrations were measured by ultra performance liquid chromatography (Waters ACQUITY UPLC H-Class System). Plasma OxS was measured using a thiobarbituric acid-reducing substance (TBARS) assay (Cayman Chemical, Ann Arbor, MI, USA), and an 8-Iso-Prostaglandin-F2α assay (Cell Biolabs Inc., San Diego, CA, USA).

#### 2.5.2. Mitochondrial Fuel Oxidation

This was measured by gas-exchange using calorimetry (Vmax Encore metabolic cart, CareFusion Inc., San Diego, CA, USA). Mitochondrial fatty-acid and glucose oxidation were also calculated [24], and expressed both as per kilogram bodyweight and per kilogram lean mass.

#### 2.5.3. Protein Isolation and Immunoblot Analyses

These were performed in the muscle biopsy samples obtained from participants in the study. Briefly, total protein was isolated from the muscle biopsy tissue using ice-cold 1X Cell Signaling lysis buffer (20 mM Tris-HCl (pH 7.5), 150 mM NaCl, 1 mM Na_2_EDTA, 1 mM EGTA, 1% Triton, 2.5 mM sodium pyrophosphate, 1 mM beta-glycerophosphate, 1 mM Na_3_VO_4_, 1 µg/mL leupeptin) and 1 mM PMSF. Protein concentration was determined by Pierce^TM^ BCA Protein Assay Kit (Thermo Scientific). The proteins were separated by SDS-PAGE followed by electrotransfer on the polyvinylidene difluoride membrane. The membranes were blocked with 5% *w/v* nonfat dry milk (Cell signaling technology) and probed using antibodies against PGC1α, PPARα, SIRT3, LC3A/B, PINK1, and β-actin, followed by a horseradish peroxidase-conjugated secondary antibody (cell signaling technology). Bands were visualized using the SuperSignal^TM^ West Dura Extended Duration Substrate (Thermo Scientific) on autoradiography film. Quantification of the immunoblot band intensity was performed using grayscale measurements with ImageJ 1.51j8 software. The expression of proteins of interest were normalized with respective β-actin.

#### 2.5.4. Tracer Studies (at 0 w and 12 w Only)

(a) Muscle protein breakdown rate:

Stable isotope preparation: sterile [^2^H_3_]-3-methylhistidine was purchased from Cambridge Isotopes Laboratories (Andover, MA, USA), dissolved in saline, and infused after sterility testing.

Isotopic Analyses: serum isotope enrichment of 3-methylhistidine was measured by liquid-chromatography tandem mass-spectrometry after conversion into its DANS [5-(dimethylamino)-1-napthalene sulfonamide] derivative, and analyzed using a Synergi Fusion-RP 2.5µ 100 × 2.0 mm column (Phenomenex, Torrance, CA) on a triple quadrupole mass-spectrometer (TSQ Vantage; Thermo Scientific, San Jose, CA, USA). Ions *m*/*z* 403 and 406 to product ion *m*/*z* 170 for 3-methylhistidine and [^2^H_3_]-3-methylhistidine, were monitored. Instrumental control, data acquisition and analysis were performed by the Xcalibur (version 2.1) software package (Thermo Scientific, San Jose, CA, USA). Serum 3-methylhistidine concentration was measured in the basal sample by an isotope dilution method using [^2^H_3_]-3-methylhistidine as an internal standard. The samples were then analyzed as described above.

Calculations: all kinetic measurements were performed under steady state conditions.

Moreover, 3-methylhistidine (3MH) flux, an index of myofibrillar protein breakdown rate, was calculated using the steady-state equation 3-methylhistidine flux = F × [(Iei/Iep) − 1], where F is the isotope infusion rate, Iei is the isotopic enrichment of the infusate in mol percent excess, and Iep is the isotopic enrichment at steady state.

Muscle protein breakdown rate (MPBR): because 3-MH is released only from the breakdown of myofibrillar protein present predominantly in skeletal muscle, its flux is used to estimate muscle protein breakdown rate based on a 3-MH concentration of 3.64 µmol/g of muscle protein. MPBR = 3 MH flux/3.64.

(b) Rates of appearance of glucose, glucose production rate, gluconeogenesis, and glycogenolysis

Stable isotope preparation: sterile and pyrogen free deuterium oxide (^2^H_2_O), ^2^H and [6,6-^2^H2] glucose was purchased from Cambridge Isotopes Laboratories (Andover, MA). Moreover, [6,6-^2^H2] glucose was dissolved in isotonic water, filtered, and infused after sterility testing as described earlier.

Isotopic Analyses: the Isotopic enrichment of [6,6-^2^H_2_] glucose was measured by gas-chromatography mass-spectrometry (6890/5973 Agilent Technologies, Wilmington, DE, USA) using the pentaacetate derivative. The incorporation of deuterium in glucose from ^2^H_2_O was determined using the average deuterium enrichment in glucose carbons 1, 3, 4, 5 and 6 [25]. Deuterium enrichment in plasma water was determined by Isotope Ratio Mass Spectrometry (Delta^+^XL isotope ratio mass spectrometry, Thermo Finnigan, Bremen, Germany).

Calculations: all kinetic measurements were performed under steady state conditions.

Rate of appearance of glucose (Ra glucose) was calculated during steady state from the M+2 enrichment of [6,6-^2^H_2_]glucose in plasma using the steady-state equation: 

Ra glucose = F × [(Iei/Iep) − 1]

Rate of glucose production (mg/kg/min) = Ra glucose − exogenous glucose tracer infused

Fractional gluconeogenesis (i.e., gluconeogenesis as a fraction of glucose Ra) was calculated as follows:

Gluconeogenesis% = [(M + 1) (^2^H) (*m*/*z* 170/169)/6]/IE^2^H_2_O, where (M + 1)(^2^H) (*m*/*z* 170/169) is the M + 1 enrichment of deuterium in glucose measured using *m*/*z* 170/169 and ‘6’ is the number of ^2^H labeling sites on the *m*/*z* 170/169 fragment of glucose and IE^2^H_2_O is the deuterium enrichment in plasma water.

Rate of gluconeogenesis (mmol/kg/min) = Ra glucose x gluconeogenesis%, was calculated as the product of total glucose appearance rate and fractional gluconeogenesis.

Rate of glycogenolysis (mmol/kg/min) = glucose production − gluconeogenesis, was calculated by subtracting the rate of gluconeogenesis from the glucose production rate.

#### 2.5.5. Physical Function

Physical function was assessed using simple validated measures, which can be easily performed in the clinical outpatient setting, and included gait speed (with the participant walking comfortably at own pace), chair-rise test (time taken to stand up and sit down as quickly as possible 10 times from a chair with arms folded across the chest), forearm grip strength (done using best of 3 readings using a Jamar dynamometer), and exercise capacity (as distance walked in 6-min while walking as quickly as possible).

#### 2.5.6. Cognitive Function

Cognition was measured as Montreal cognitive assessment (MoCA), trail making tests A and B, verbal fluency test (VFT), and the digital symbol substitution test (DSST). These are standard validated tests which can be used in the clinical outpatient setting.

#### 2.5.7. Insulin Resistance

Fasted plasma glucose concentrations were measured with an automated glucose analyzer (Yellow Springs Instrumentation, Yellow Springs, OH, USA), and insulin concentrations were measured using an ELISA kit (Mercodia, Uppsala, Sweden). Insulin resistance was calculated as HOMA-IR, as reported by us previously [13].

#### 2.5.8. Plasma Biomarkers of Inflammation, Endothelial Function, and DNA Damage

These were measured using a high-sensitivity interleukin 6 (IL-6) human, tumor-necrosis factor alpha (TNFα), human soluble intercellular adhesion molecule 1 (ICAM-1), soluble vascular cell adhesion molecule (VCAM-1) and E-selectin human ELISA kits (Invitrogen Waltham, MA, USA; Thermo Scientific Inc., Frederick, MD, USA), and high-sensitivity human C-reactive protein (CRP) Quantikine ELISA kit (R&D Systems, Minneapolis, MN, USA). DNA/RNA Oxidative damage was measured a high-sensitivity 8-hydroxy-2-deoxyguanosine (8-OHdG) ELISA Kit (Cayman Chemicals, Ann Arbor, MI, USA).

#### 2.5.9. Body Composition and Anthropometry

Total body and truncal fat were measured by DEXA scans. Height and weight were measured using a stadiometer and standardized, calibrated weighing scale, and used to calculate body mass index. Waist circumference was measured using a tape measure, and the same sites were used for repeat measurements. Liver fat was assessed before and 12 weeks after GlyNAC supplementation by magnetic resonance spectroscopy using standardized protocols.

### 2.6. Statistics

Baseline characteristics are summarized by means with standard deviations or frequencies with percentages. Controls were matched to PWH in a 1:1 ratio by age, sex, and BMI. A paired t-test was used to compare PWH and matched controls at baseline. A general linear mixed model was used to test for changes in outcomes across time points for PWH. The model included fixed effects for study time (discrete) and the matrix of correlated residuals assumed an unstructured format. The mixed model is used to estimate means with 95% confidence intervals and test the null hypothesis that there is no difference in means between time points. All hypothesis tests were assessed at the 0.05 level (two-sided). The *p*-values were not adjusted for multiple hypothesis testing due to the small sample size of this pilot study.

## 3. Results

### 3.1. Adverse Effects or Participant Withdrawals

No adverse effects were reported, and no participants withdrew participation.

### 3.2. HIV Parameters

All patients were on antiretroviral treatment and had suppressed HIV viral loads (<20 copies/mL). Their CD4 (cluster of differentiation 4) counts ranged from 350 to 1276 (mean 726). All patients were on stable antiretroviral treatments (7 of 8 were on Reyataz and Norvir (with a varying additional third drug which included Epzicom, Abacavir, Atripla, Truvada, or Viread), and 1 patient was on monotherapy with Triumeq).

### 3.3. Plasma Biochemistry

All participants had normal thyroid stimulating hormone, free thyroxine, testosterone, hemoglobin, renal and liver functions (Table 1). Glycosylated hemoglobin was in the nondiabetic range. Supplementation with GlyNAC was well tolerated without an increase in creatinine, ALT or AST (measured every 4 weeks), and there was a trend toward decrease in plasma alanine transaminase concentrations (*p* = 0.08). There were no changes in total, low-density lipoprotein cholesterol (LDLc) or high-density lipoprotein cholesterol (HDLc), triglyceride concentrations, or HbA1c. Interestingly estimated glomerular filtration rate (GFR) improved significantly (*p* = 0.004).

### 3.4. Glutathione and Oxidative Stress

**Figure 1:**
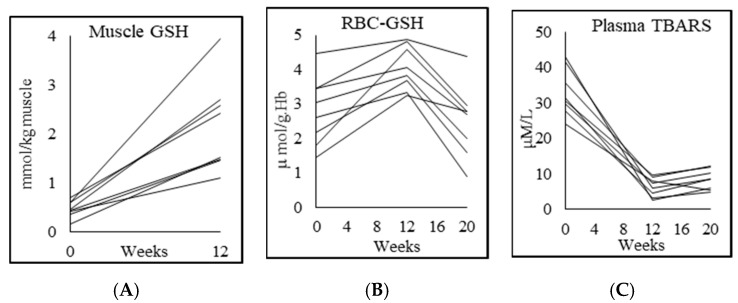
Individual participant data showing the effect of supplementing PWH with 12 weeks of Glycine and N-acetylcysteine and 8 weeks of withdrawal (as applicable). Shown here are (**A**,**B**) total GSH concentrations in muscle and RBC respectively and (**C**) plasma TBARS concentrations.

PWH had GSH deficiency in muscle and RBC, and significantly higher plasma OxS (Table 2; Figure 1). GlyNAC supplementation for 12 weeks increased muscle GSH by 340% and RBC-GSH by 46%, while TBARS fell by 81%, and F2-isoprostanes fell by 80%. The improvements in these outcomes receded after stopping GlyNAC supplementation for 8 weeks.

### 3.5. Mitochondrial Fuel Oxidation

**Figure 2:**
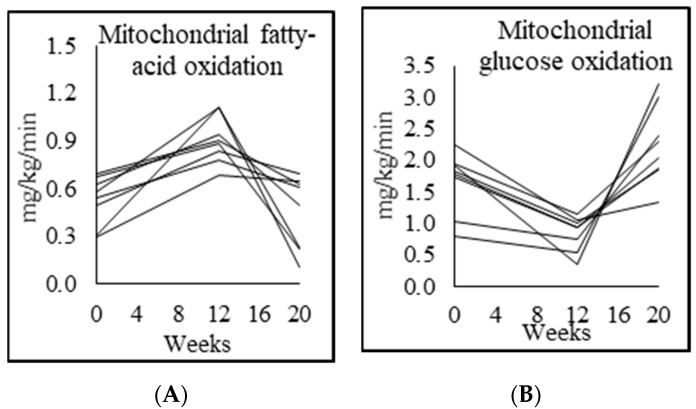
Individual participant data showing fasted mitochondrial (**A**) fatty-acid oxidation and (**B**) glucose oxidation.

Compared to controls, fasted unsupplemented PWH at baseline had significantly higher respiratory quotient, and had lower MFO and higher mitochondrial glucose oxidation (MGO). GlyNAC supplementation led to a significant increase in MFO, and decrease in MGO. However, these improvements were lost after withdrawal of GlyNAC for 8 w with a return of MFO and MGO to pre-supplementation values (Table 3; Figure 2). Interestingly, there was no change in total energy expenditure with GlyNAC supplementation or withdrawal, suggesting that the fuel shift between fatty-acids and glucose did not impact overall energy expenditure. 

### 3.6. Tracer Kinetic Data and Glucose Metabolism

**Figure 3:**
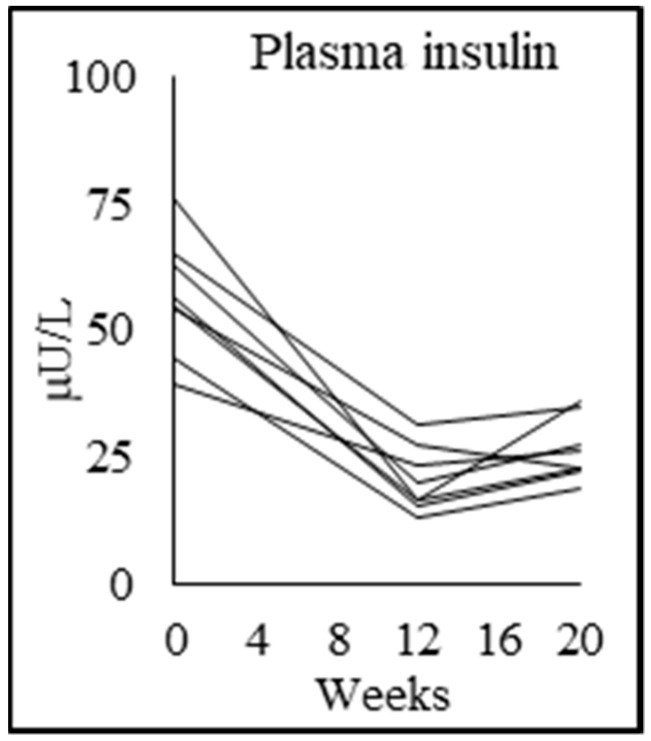
Individual participant data showing fasted plasma insulin concentrations.

PWH at baseline had significantly higher MPBR, and this declined significantly with GlyNAC supplementation (Table 4). PWH had significantly higher fasting plasma glucose, higher insulin concentrations, and insulin resistance. These parameters improved with GlyNAC, and receded after stopping GlyNAC (Table 4; Figure 3). There were no baseline differences between controls and PWH for gluconeogenesis, glycogenesis and glucose production rate, and these were not affected by GlyNAC supplementation. The implications of these changes on cognitive function is addressed in the discussion. 

### 3.7. Inflammation, Endothelial Function, and Genotoxicity

**Figure 4:**
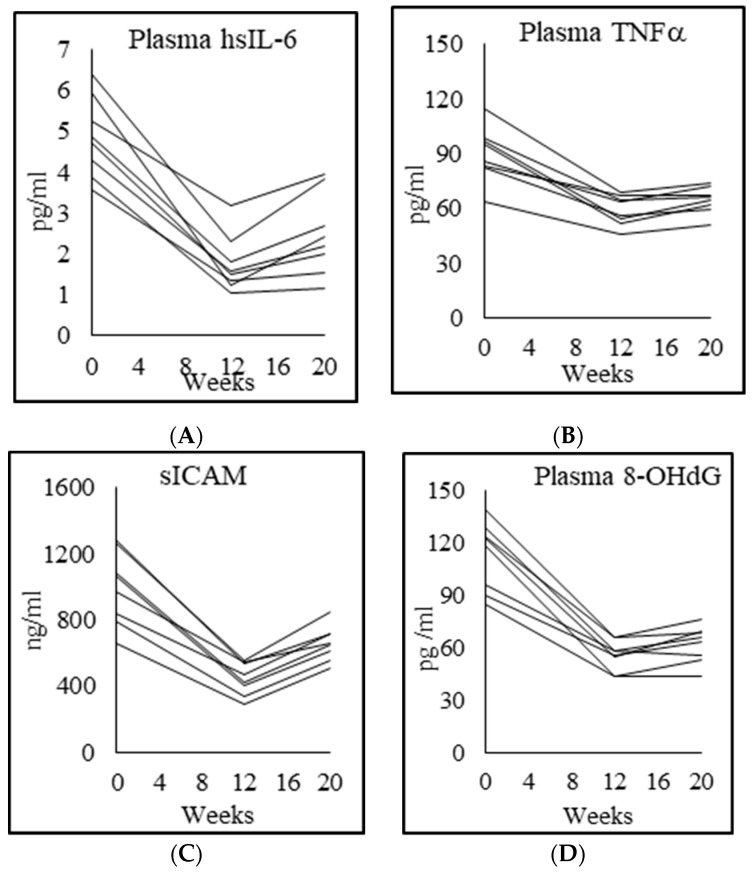
Individual participant data showing plasma concentrations of (**A**) interleukin 6 (IL-6); (**B**) tumor-necrosis factor alpha (TNFα); (**C**) soluble intercellular adhesion molecule 1 (sICAM1); and (**D**) 8-hydroxy-2-deoxyguanosine (8-OHdG) concentrations.

Inflammation: PWH had significantly higher levels of proinflammatory cytokines high-sensitivity hsIL6, hsCRP and TNFα compared with matched controls at baseline (hsIL6 518% higher; hsCRP 450% higher; TNFα 166% higher). After 12 weeks of GlyNAC supplementation, plasma concentrations of these elevated proinflammatory biomarkers declined significantly (hsIL6 65% lower, hsCRP 54% lower, and TNFα levels 34% lower). After stopping GlyNAC supplementation for 8 weeks, levels of these proinflammatory biomarkers began to increase (hsIL6 by 42%, hsCRP by 15%; TNFα by 9%), but these levels were still significantly lower than their pre-supplementation baseline values (hsIL6, *p* = 0.000; hsCRP, *p* = 0.00; TNFα, *p* = 0.00), suggesting that the beneficial effects of GlyNAC supplementation on lowering inflammation persist even 8 weeks after stopping GlyNAC (Table 5; Figure 4).

Endothelial function: PWH had significantly higher levels of sICAM1, sVCAM1 and E-selectin compared with controls (sICAM1 172% higher; sVCAM1 162% higher; E-selectin 123% higher). After 12 weeks of GlyNAC supplementation, there was a striking decline in endothelial dysfunction (sICAM1 55% lower), sVCAM1 41% lower; E-selectin (35% lower). After stopping GlyNAC supplementation for 8 weeks, levels of these biomarkers of endothelial dysfunction increased (sICAM1 48% increase; sVCAM1 28% increase; E-selectin 27% increase), but these values remained significantly lower than their pre-supplementation baseline values suggesting that the effects of GlyNAC supplementation on endothelial function persisted even 8 weeks after stopping GlyNAC (Table 5; Figure 4).

Genotoxicity: plasma levels of 8-hydroxydeoxyguanozine (8-OHdG), which is a marker of genomic damage was significantly higher in PWH than matched controls, declined significantly with GlyNAC supplementation, and increased after stopping GlyNAC for 8 weeks (Table 5; Figure 4).

### 3.8. Physical Function

**Figure 5:**
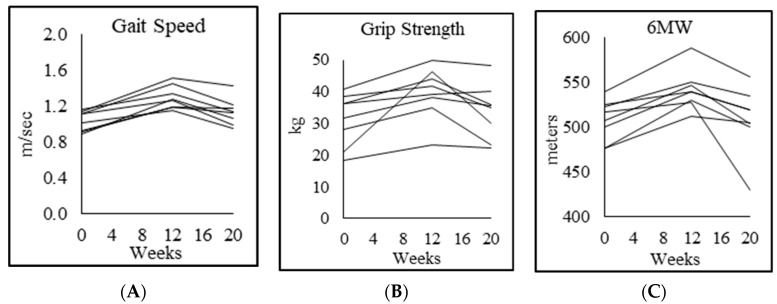
Individual participant data showing (**A**) gait speed, (**B**) forearm grip strength (dominant), and (**C**) rapid 6-min walk test (6MW).

Compared to controls, PWH at baseline had significant 23% lower gait speed, 32% lower forearm grip strength in the dominant hand and 35% lower in the nondominant hand, 52% higher chair-rise test score, and 21% lower rapid 6-min walk test score. Moreover, 12 weeks of GlyNAC supplementation led to striking improvements in gait speed, forearm grip strength (25% increase in dominant hand; 29% nondominant), 20% improvement in chair-rise test score, and 6-min walk test score increased by 7%. All values approached that in unsupplemented controls, with no differences between controls and PWH in gait speed (*p* = 0.6), forearm grip strength (dominant hand *p* = 0.14; nondominant hand *p* = 0.2) and chair-rise test (*p* = 0.1), but the 6-min walk test still remained significantly lower than controls despite supplementation (*p* < 0.01). Stopping GlyNAC supplementation for 8 weeks led to a loss of accrued benefits in gait speed, forearm grip strength (dominant and nondominant hands), chair-rise test, and 6-min walk test, suggesting that continued supplementation with GlyNAC may be necessary for preserving physical function. (Table 6; Figure 5).

### 3.9. Cognition

**Figure 6:**
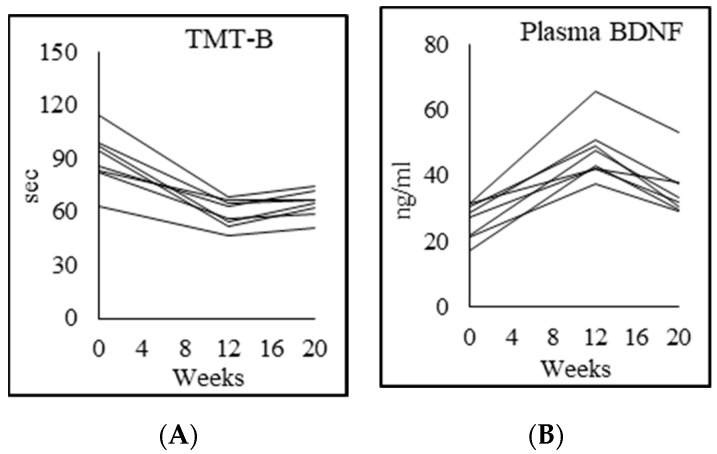
Individual participant data showing (**A**) Trails-B test values and (**B**) fasting plasma BDNF concentrations.

Compared to controls, PWH at baseline had significantly lower cognitive function as assessed by the following battery of tests: Montreal cognitive assessment (MoCA 25% lower), Trails A test (81% higher), Trails B test (118% higher), DSST %completion (30% lower), DSST %accuracy (4% lower), and VFT (30% lower) (Table 7; Figure 6). After 12 weeks of GlyNAC supplementation there were striking improvements in MoCA (18%), Trails A test (26%), Trails B test (41%), DSST % completion (19%), DSST % accuracy (3%), and VFT (20%). Post-supplementation MoCA test (*p* < 0.05) and Trails A test (*p* < 0.05) remained significantly lower than controls, but there were no significant differences in Trails B test (*p* = 0.17), cognitive flexibility (*p* = 0.69), DSST %completion (*p* = 0.083), DSST %accuracy (*p* = 0.41), and VFT (*p* = 0.23) between controls and GlyNAC-supplemented PWH. Stopping GlyNAC supplementation for 8 weeks was associated with a non-significant decline in accrued benefits in MoCA, Trails B test, DSST %completion (*p* = 0.47), DSST %accuracy (*p* = 0.71), VFT (*p* = 0.28), while the Trails A test continued to improve (*p* = 0.068). We also measured and found that plasma concentrations of brain-derived neurotropic factor (BDNF, an index of memory) was significantly lower than controls in unsupplemented PWH, and improved significantly with 12 w of GlyNAC, but declined on stopping GlyNAC for 8 w, suggesting that the improvements in cognitive function are associated with improvements at a biological level as represented by BDNF. 

### 3.10. Body Composition

**Figure 7:**
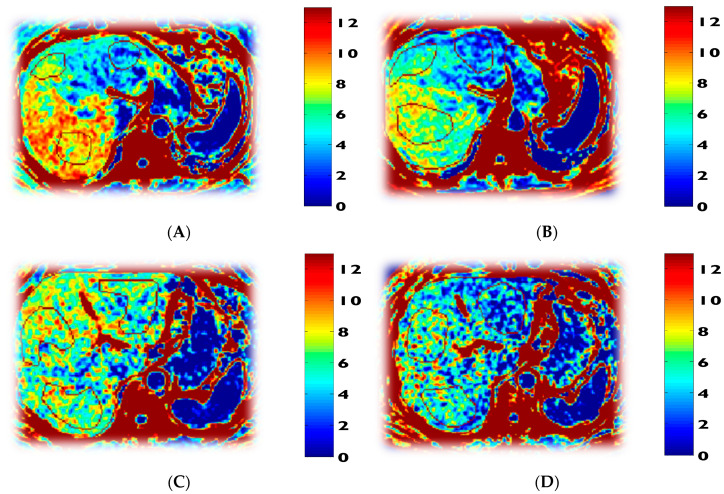
Effect of supplementing GlyNAC on liver magnetic resonance spectroscopy (MRS) in 2 participants with non-alcoholic fatty-liver disease (NAFLD). The panels show liver MRS data of % fat-fraction in 2 participants done before and 12 w after receiving GlyNAC supplementation: (**A**) participant 1 before GlyNAC supplementation; (**B**) participant 1 after GlyNAC supplementation; (**C**) participant 2 before GlyNAC supplementation; (**D**) participant 2 after GlyNAC supplementation.

PWH were matched to controls for body mass index (BMI), and hence there were no differences between the two groups at baseline for BMI, but PWH had significantly higher truncal fat (32% higher) and 9% higher waist-to-hip ratio suggesting greater central abdominal fat accumulation (Table 8; Figure 7). After PWH received 12 weeks of GlyNAC supplementation there was a striking decline in BMI, total fat (decreased by 2.3 kg), truncal fat (decreased by 1.6 kg), waist circumference (decreased by 7.3 cm), and waist:hip ratio, and comparison with controls showed no significant differences in truncal fat (*p* = 0.07), waist circumference (*p* = 0.9), or WHR (*p* = 0.6). After stopping GlyNAC supplementation for 8 weeks, the waist circumference increased by 2.3%, and WHR increased 2.3%, suggesting that central fat tended to reaccumulate after GlyNAC withdrawal, but these values were lower than the pre-supplementation values suggesting the benefits of 12 w of GlyNAC supplementation on central fat persisted 8 w after stopping GlyNAC. There were no differences in lean mass between PWH and controls at baseline, and there were no significant changes with 12 w of GlyNAC supplementation. PWH had significantly higher systolic and diastolic blood pressure, which decreased significantly with GlyNAC. Although all participants had magnetic resonance spectroscopy scans to assess liver fat, only 2 participants had elevated liver fat at the basal scan, and the liver fat fraction decreased by 40% with GlyNAC supplementation. 

### 3.11. Molecular Regulation of Energy Metabolism, Autophagy, and Mitophagy

**Figure 8:**
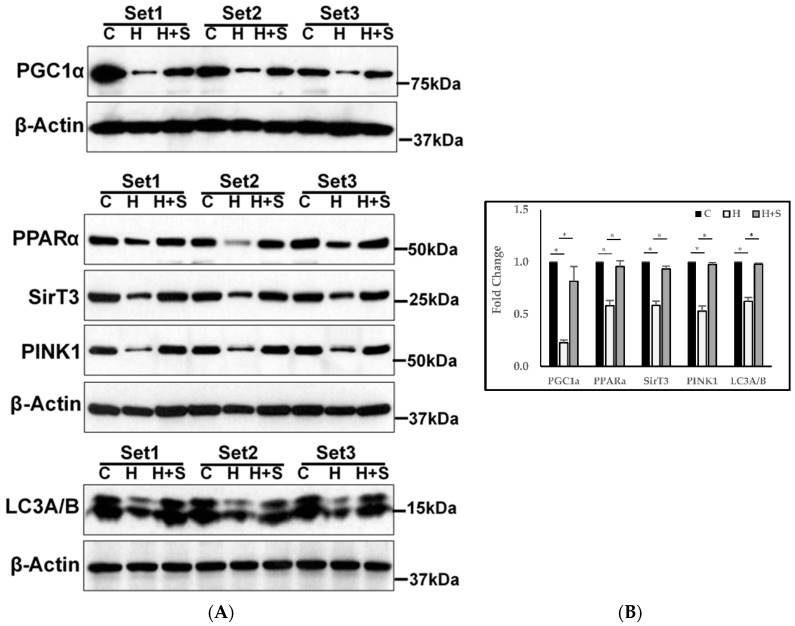
Effect of supplementing GlyNAC improves molecular regulation of mitochondrial energy metabolism, mitophagy, and autophagy in PWH. (**A**) immunoblots; (**B**) quantification of immunoblots using Image-J software. C = Control; H = PWH pre-supplementation; H + S = PWH 12 w after receiving GlyNAC supplementation. For all blots shown below, C vs. H = *p* < 0.05; H vs. H + S = *p* < 0.05; * = *p* < 0.05.

PWH had significantly lower protein expression of regulators of mitochondrial energy metabolism (PGC1α, PPARα, SIRT3), autophagy (LC3A/B), and mitophagy (PINK1), and these improved significantly after GlyNAC supplementation (Figure 8). Participants did not undergo a biopsy after withdrawal of GlyNAC.

## 4. Discussion

This study highlights the impact of GlyNAC supplementation on several key defects associated with premature aging in PWH. The salient findings are that: (1) Compared to age, gender and BMI-matched HIV uninfected controls, PWH had (a) GSH deficiency and elevated OxS; (b) impaired MFO and MGO; (c) abnormally low protein expression of key molecular regulators of energy-metabolism, autophagy and mitophagy; (d) cognitive impairment; (e) lower physical function; (f) elevated inflammation and endothelial dysfunction; (g) insulin resistance with elevated fasted glucose and insulin concentrations, but without an increase in fasting rates of gluconeogenesis, glycogenolysis, or glucose production; (h) higher total body fat, truncal fat, waist circumference, body weight, BMI, and blood pressure; (i) increased muscle protein breakdown; (2) GlyNAC supplementation for 12 weeks improved these defects; (3) Several accrued benefits receded 8 weeks after stopping GlyNAC. These findings could have implications for premature aging in PWH, and are discussed below.

### 4.1. Effect of GlyNAC on GSH Deficiency and OxS

We showed earlier that in PWH between 50–60 years of age, deficiency of glycine and cysteine account for diminished GSH synthesis and concentrations [13]. The current trial found that compared to controls, PWH in the 45–65 years age range in this study had severe intracellular GSH deficiency and elevated OxS, and this could be improved with 12 weeks of GlyNAC supplementation (using doses identical to our prior study). However, accrued benefits reverted to pre-supplementation values after stopping GlyNAC, suggesting that continued supplementation is necessary to maintain optimal GSH supply.

### 4.2. Effect of GlyNAC on Mitochondrial Fuel Oxidation and Molecular Regulation of Energy Metabolism

We previously reported that GSH adequacy is critically important for optimal fasted MFO [23]. Key findings in the current study are that compared to controls, PWH had significantly lower fasted MFO and higher MGO, and that these mitochondrial defects were corrected with GlyNAC supplementation. In the fasted state, fatty-acids (not glucose) are the primary fuel of choice for mitochondrial energy generation in non-brain tissues. PWH in this study had impaired fasting MFO and elevated fasting MGO, indicating an abnormal reversal in expected pattern of fasted mitochondrial fuel oxidation. Supplementing GlyNAC for 12 weeks corrected these defects by increasing MFO and lowering MGO, but withdrawal of GlyNAC supplementation for 8 weeks resulted in a loss of these benefits, and both fasted MFO and MGO returned to pre-supplementation values. In a prior study we found GlyNAC supplementation for a short duration of 2 weeks improved MFO and MGO [13]. The current study extends and confirms these findings by showing that 12-week duration of GlyNAC supplementation not only improves MFO and MGO but restores these values to approximate matched uninfected controls suggesting reversal of mitochondrial dysfunction. Furthermore, this improvement argues against a tachyphylaxis effect over a 12-week supplementation, and the loss of mitochondrial benefits on stopping GlyNAC suggests that continued supplementation with GlyNAC is needed for maintaining mitochondrial benefits. In addition to these whole-body findings, fasted PWH also had abnormalities in the molecular regulators of energy metabolism in skeletal muscle, with lower protein expression of PGC1α, PPARα, and SIRT3. PGC1α is a transcriptional co-activator, and considered a master regulator of mitochondrial energy metabolism including mitochondrial biogenesis, oxidative phosphorylation, fatty-acid oxidation, and expression of reactive oxygen species detoxifying antioxidant enzymes [26,27,28]. Therefore, impaired expression of PGC1α in PWH could represent a key defect contributing to premature aging, and reversible with GlyNAC supplementation. PPARα is widely implicated in the upregulation of mitochondrial uptake and oxidation of fatty-acids, and downregulation of OxS, inflammation, insulin resistance, metabolic syndrome and fatty liver. Hence decreased expression of PPARα in PWH is relevant, because PWH are reported to have an increased prevalence of impaired MFO, metabolic syndrome, inflammation, insulin resistance, endothelial dysfunction, and cardiovascular disease [14,15,16,21,22,23,24,25,26,27,28,29,30]. Mitochondrial sirtuins are NAD^+^ dependent enzymes, which are conserved from bacteria to humans. NAD^+^ is of vital importance in energy generation in mitochondria, and has been implicated in increasing longevity in yeast, flies, worms, and mice [31,32]. Sirt3 is also involved in combating reactive oxygen species, oxidative stress and maintaining mitochondrial integrity and MFO [33,34,35]. Hence, the observation that GlyNAC supplementation led to recovery of decreased Sirt3 expression in muscle of PWH could be another pathway for improving multiple metabolic and mitochondrial defects in PWH. Collectively, the recovery of PGC1α, PPARα, and SIRT3 due to GlyNAC supplementation could account for the diverse and multiple mitochondrial and metabolic benefits seen in PWH in this study.

### 4.3. Effect of GlyNAC on Rate of Muscle Protein Breakdown

This study found that compared to controls, PWH with premature aging have elevated muscle protein breakdown rate (MPBR), and that GlyNAC supplementation lowered the MPBR to levels seen in controls. This finding could shed light on the mechanism/s contributing to muscle loss in PWH, and provide leads for novel strategies to combat sarcopenia. Maintenance of muscle mass requires a balance between muscle protein synthesis and breakdown. PWH have a high prevalence of muscle loss [36], but correcting this is challenging. Interventions to target sarcopenia in PWH have primarily focused on boosting muscle protein synthesis via anabolic strategies including dietary protein supplementation, growth hormone, and testosterone. However, success with these anabolic strategies has been limited, because potential increases in muscle synthesis could have been offset by increased muscle breakdown. Although a decrease in muscle protein breakdown suggests that muscle mass could increase, this was not seen in our study, and can be explained by understanding that increasing muscle mass may depend on both increasing muscle protein synthesis, and decreasing muscle breakdown. While GlyNAC supplementation lowered MPBR, our study did not include an anabolic stimulus to simultaneously boost muscle protein synthesis, and we speculate this to be the reason for lack of increase in muscle mass. Nevertheless, the reduction in MPBR is an important finding which holds implications for improving muscle health and combating sarcopenia in PWH, and warrants further investigation.

### 4.4. Effect of GlyNAC on Muscle Strength and Exercise Capacity

Declining physical function is a key defect associated with premature aging in PWH, and manifests as lower gait speed, muscle strength, and exercise capacity [9]. The mechanistic underpinnings of declining physical function in PWH with premature aging are not well understood. Meta-analyses in geriatric populations have strongly linked gait speed to survival [37], and PWH aged > 40 years are reported to have increased functional decline, especially in gait speed [8]. Supplementing GlyNAC in PWH improved physical function and improved exercise capacity, thereby ‘reversing’ premature physical aging. However, these benefits declined on stopping GlyNAC suggesting that continued supplementation is necessary to maintain its benefits in physical function. Overall, these results are encouraging because interventions to improve physical functioning, muscle strength and exercise capacity in PWH are limited.

### 4.5. Effect of GlyNAC Supplementation on Improving Insulin Resistance

GlyNAC supplementation for 12 weeks significantly lowered insulin resistance by 69%. The striking magnitude of this response is supported by our prior studies in HIV and aging, where PWH with premature aging receiving 2 weeks of GlyNAC supplementation (at the same dose in PWH of similar age range) improved insulin sensitivity by 31% (measured by the gold-standard hyperinsulinemic-euglycemic clamp technique) [13], geriatric (uninfected) humans supplemented with GlyNAC for 2 weeks experienced 40% reduction in insulin resistance (measured as HOMA-IR) [23], and aged mice supplemented with GlyNAC reversed glucose intolerance and insulin resistance and matched levels in young control mice [23]. Multiple factors could account for this marked reduction in insulin resistance in PWH in this study. For example, elevated OxS could have induced insulin resistance by impairing insulin signaling and decreasing Glut4 transcription as reported in other studies [38,39], and reductions in OxS in our study could have improved insulin resistance. Insulin resistance is also linked to impaired MFO [40,41], and we have previously reported that improving impaired MFO with GlyNAC is associated with striking improvements in insulin resistance [23]. Therefore, multiple factors stemming from GSH deficiency, elevated OxS and impaired MFO could have contributed to the striking increase in insulin resistance in PWH, and the reversal of these key defects with GlyNAC supplementation lowered insulin resistance.

### 4.6. Effects of GlyNAC on Cognitive Decline

An important discovery in this study was the impact of supplementing GlyNAC on reversing cognitive decline in PWH. This improvement appears to be real, and not due to practice effects. This is because if the improvement was due to practice effects, there should have been a progressive improvement with each successive test—although cognition improved when tested a second time after GlyNAC supplementation (at 12 weeks), tests either deteriorated or did not improve further when tested a 3rd time after GlyNAC withdrawal (at 20 weeks). In addition, changes in cognitive performance were mirrored by an increase in plasma concentrations of brain-derived neurotropic factor (BDNF), involved in long-term memory [42] with GlyNAC, and a decrease on withdrawing GlyNAC. The improvement of BDNF levels with GlyNAC and its decline after withdrawing GlyNAC tracks with performance on the cognitive testing, and because BDNF is a biological marker, it is not influenced by any practice effect. Why does GlyNAC improve cognition? Since the primary fuel for the brain is glucose, valuable clues come from paradoxical observations that prior to GlyNAC supplementation, whole-body glucose oxidation was higher, but paradoxically cognition was lower; after GlyNAC supplementation whole-body glucose oxidation decreased, but paradoxically cognition improved. The brain needs glucose to generate energy in both the fasting and fed states, whereas non-brain organs and tissues such as liver, muscle, heart, etc., primarily utilize fatty-acids to generate energy in the fasting state. Under physiological fasting conditions, plasma glucose is supported by endogenous glucose production. Prior to supplementing with GlyNAC, endogenous glucose production in PWH was not higher relative to controls, suggesting that the supply of glucose was not increased. The higher whole-body glucose oxidation in PWH prior to GlyNAC indicates that non-brain organs were substituting some glucose instead of fat as an energy substrate in the fasting state because mitochondrial fatty acid oxidation was impaired secondary to GSH deficiency. That is, they were competing with the brain for glucose effectively ‘stealing’ glucose away from the brain. This leaves less glucose available for the brain which results in impaired cognition. After GlyNAC supplementation, GSH supply and availability increased resulting in improved MFO in non-brain tissues. This reduced their need for glucose as an energy substitute, hence, more glucose was available for the brain, and cognition improved. This observation could help explain why treated PWH have decreased brain glucose uptake on FDG-PET (fluorodeoxyglucose positron emission tomography) scans [43]. Another contributor to cognitive decline in PWH could be the severe insulin-resistance which could have limited glucose entry into the brain, and the improvement of insulin-resistance with GlyNAC could have facilitated improved brain glucose uptake and thereby improved cognition in PWH in this study. Since cognitive impairment is a significant comorbidity in PWH and there is a limitation of effective interventions, the findings of our study offer an exciting lead on combating cognitive decline in PWH, and warrants additional investigation.

### 4.7. Effect of GlyNAC on Total Body Fat, Waist Circumference, and Liver Fat

Impaired MFO and increased MGO are risk factors for weight gain [44]. Increases in abdominal and total obesity are a real problem in PWH. We found that supplementing GlyNAC in PWH was associated with a significant reduction in BMI, total body fat, truncal fat mass, a striking decrease in waist circumference of 7.3 cm, and lowered waist-to-hip ratios without any change in the hip circumference, which suggests that the fat loss preferentially occurred due to loss of abdominal fat. Although the study did not focus on non-alcoholic fatty-liver disease (NAFLD), hepatic imaging revealed subclinical NAFLD in 2 of 8 participants studied, and the liver fat-fraction decreased by 40% with GlyNAC supplementation in these 2 participants (Figure 3). These data are consistent with our earlier publication where we found a similar effect of GlyNAC on significantly decreasing in total body fat and liver fat in aged mice in association with improving GSH deficiency and mitochondrial dysfunction [23]. The available evidence suggests that impaired mitochondrial fatty-acid oxidation could promote fat storage and result in elevated fat accumulation, and that GlyNAC supplementation could lower total body fat, truncal fat, and liver fat by improving MFO. Furthermore, the presence of increased waist circumference, insulin resistance, elevated blood pressure, and triglyceride levels all suggest that PWH in this study also had metabolic syndrome, which was improved with GlyNAC. Collectively these data suggest GlyNAC supplementation could play an important role on lowering elevated total body fat, truncal fat, liver fat content, and metabolic syndrome, and warrants additional investigation.

### 4.8. Effect of GlyNAC on Inflammation, Endothelial Function, and Cardiovascular Risk

Elevated inflammation and endothelial dysfunction occurs in PWH [14,15,16], but their mechanistic origins are not well understood. We found that GlyNAC supplementation significantly improved plasma concentrations of key biomarkers of inflammation (IL-6, TNFα, hsCRP) and endothelial dysfunction (sICAM1, sVCAM1and E-selectin), but accrued benefits receded after stopping GlyNAC supplementation. Because elevated inflammation, endothelial dysfunction, insulin-resistance and waist-circumference are linked to cardiovascular disease, improvement in these parameters suggest the possibility of lowering cardiovascular risk with GlyNAC supplementation in PWH, and supports the need for additional studies.

### 4.9. Effect of GlyNAC on Genomic Damage

Increased oxidative genomic (DNA) damage has been reported in younger PWH < 60 years [45], and has been linked to accelerated aging [17,46] and cognitive impairment [47]. Mechanisms contributing to genomic damage are unclear, and interventions lacking. In this study, GlyNAC supplementation led to significant decline in genomic damage (measured as plasma concentrations of 8-OHdG), but benefits receded 8 weeks after stopping GlyNAC supplementation. This improvement in genotoxicity is another piece of evidence supporting potential reversibility of premature aging in PWH, and correlates with cognitive improvement. These data suggest that GlyNAC supplementation could attenuate genomic damage in PWH.

### 4.10. Hallmarks of Aging

The field of geroscience has identified nine discrete hallmarks of aging which underlie and promote aging in humans [48]. Although these have not been studied in PWH, we find in our study that PWH had abnormalities in at least five out of nine hallmarks of aging (mitochondrial dysfunction, genomic instability, dysregulated nutrient sensing (depicted as insulin resistance and abnormal SIRT3 expression), altered intercellular communication (depicted as inflammation), and loss of proteostasis (abnormal autophagy and mitophagy), and these defects recover with GlyNAC supplementation for 12 weeks. These data suggest that GSH deficiency and oxidative stress could contribute to at least five aging hallmarks defects in PWH, and can be improved with GlyNAC supplementation. If this observation is confirmed to be real in future studies, it could have far-reaching implications for improving the health of PWH and also for humans with geriatric aging, and, therefore, warrants further investigation.

## 5. Conclusions

This study suggests that GSH deficiency is associated with several defects linked to premature aging in HIV patients, and provides proof-of-concept that supplementing GlyNAC improves multiple key defects associated with premature aging in PWH including GSH deficiency, oxidative stress, impaired mitochondrial fuel oxidation, elevated inflammation, endothelial dysfunction, insulin resistance, genomic damage, strength, and cognitive defects. Dietary supplementation of GlyNAC could be developed as a simple, safe, and effective nutritional strategy to improve cellular aging and health of PWH. The results of this study supports the need for future trials to assess the effects of longer durations of GlyNAC supplementation and withdrawal in PWH, as well as in healthy individuals.

## Figures and Tables

**Table 1:** Serum biochemistry before and after supplementation with glycine and N-acetylcysteine (GlyNAC). ‘Controls’ (Con) = uninfected participants not on supplementation; ‘patients with HIV (PWH): 0 weeks’ = HIV participants studied before receiving supplementation with GlyNAC; ‘PWH: 12 weeks’ = HIV participants studied 12 weeks after receiving supplementation with GlyNAC. Values are means ± SD; means are significantly different at *p* < 0.05.

Parameters	Controls	PWH: 0 weeks before Supplementation	PWH: 12 weeks after Supplementation	*p*-Value Con vs. HIV 0 w; HIV 0 w vs. HIV 12 w
Hemoglobin (g/L)	13.8 ± 1.3	14.0 ± 0.6	13.3 ± 0.8	*p* = 0.8; *p* = 0.07
Total bilirubin (mg/dL)	0.7 ± 0.3	1.2 ± 1.2	1.2 ± 1.4	*p* = 0.6; *p* = 0.8
Alanine transaminase (U/L)	24.8 ± 12.3	27.5 ± 11.1	19.9 ± 5.5	*p* = 0.7; *p* = 0.08
Aspartate transaminase (U/L)	24.3 ± 11.2	24.3 ± 3.5	21.1 ± 2.4	*p* = 1.0; *p* = 0.1
Alkaline phosphatase (U/L)	70.8 ± 30.1	87.1 ± 24.1	93.4 ± 23.6	*p* = 0.3; *p* = 0.2
Blood urea nitrogen (mmol/L)	12.8 ± 2.7	11.8 ± 3.0	9.8 ± 2.7	*p* = 0.4; *p* = 0.1
Creatinine (mg/dL)	0.9 ± 0.1	0.8 ± 0.1	0.8 ± 0.2	*p* = 0.5; *p* = 0.8
Estimated glomerular filtration rate (eGFR mL/min)	94.4 ± 7.0	92.6 ± 14.9	107.3± 20.3	*p* = 0.8; *p* = 0.004
HbA1c (%)	5.7 ± 0.4	5.8 ± 0.3	5.8 ± 0.4	*p* = 0.8; *p* = 0.5
Plasma glucose (mmol/L)	4.8 ± 0.4	5.5 ± 0.7	4.8 ± 0.6	*p* = 0.048; *p* = 0.000
Total cholesterol (mg/dL)	192.8 ± 53.5	219.5 ± 42.8	210.6 ± 54.2	*p* = 0.3; *p* = 0.6
Triglycerides (mg/dL)	91.5 ± 38.8	194.4 ± 140.7	138.9 ± 67.4	*p* = 0.1; *p* = 0.3
High density lipoprotein-cholesterol (mg/dL)	57.9 ± 14.8	41.9 ± 10.6	43.4 ± 15.5	*p* = 0.028; *p* = 0.2
Low density lipoprotein-cholesterol (mg/dL)	116.8 ± 42.6	136.9 ± 28.3	137.5 ± 41.3	*p* = 0.3; *p* = 0.8
Thyroid stimulating hormone (mIU/L)	1.9 ± 1.0	1.6 ± 0.8	1.4 ± 0.7	*p* = 0.6; *p* = 0.6
Free T4 (ng/L)	1.1 ± 0.1	1.1 ± 0.2	1.0 ± 0.1	*p* = 1; *p* = 0.5
Cortisol (mg/dL)	10.5 ± 4.8	7.5 ± 3.1		*p* = 0.2

**Table 2:** Effect of GlyNAC supplementation and withdrawal on red blood cells glutathione (RBC GSH) concentrations and oxidative stress in PWH. ‘Controls’ = unsupplemented, uninfected participants; ‘PWH: 0 weeks’ = HIV participants studied before GlyNAC supplementation; ‘PWH: 12 weeks’ = HIV participants 12 weeks after GlyNAC supplementation; ‘PWH: 20 weeks’ = HIV participants 8 weeks after stopping GlyNAC. Values are means ± SD; Means are significantly different at *p* < 0.05. BMI = body mass index; Hb = hemoglobin; GSSG = oxidized glutathione; TBARS = thiobarbituric acid reducing substances (a plasma marker of oxidative stress).

Parameters	Controls	PWH: 0 Weeks *p*-Value: HIV 0 w vs. Con	PWH: 12 Weeks *p*-Value: HIV 0 w vs. HIV 12 w	PWH: 20 Weeks *p*-Value: HIV 12 w vs. HIV 20 w
**Matching parameters:**				
Age (y)	55.0 ± 3.6	54.9 ± 4.4 *p* = 0.9		
Gender (M:F)	6:2	6:2		
Body-mass index (BMI)	28.9 ± 2.7	29.5 ± 2.3 *p* = 0.7		
**Glutathione and oxidative stress:**				
RBC-GSH (μmol/g Hb)	4.5 ± 0.6	2.8 ± 1.0 *p =* 0.002	4.1 ± 0.6 *p* = 0.003	2.5 ± 1.0 *p* = 0.001
RBC-GSSG (μmol/g Hb)	1.0 ± 1.0	0.4 ± 0.4 *p* = 0.2	0.5 ± 0.4 *p* = 0.8	1.0 ± 0.5 *p* = 0.004
Skeletal muscle GSH (mmol/kg muscle)	2.2 ± 0.8	0.5 ± 0.2 *p* = 0.000	2.2 ± 0.9 *p* = 0.001	
Plasma TBARS (μM/L)	2.7 ± 1.3	32.9 ± 6.6 *p* = 0.000	6.4 ± 2.7 *p* = 0.000	8.5 ± 2.9 *p* = 0.025
Plasma F2-isoprostane (pg/mL)	50.7 ± 3.7	267.7 ± 102.9 *p* = 0.001	53.3 ± 3.7 *p* = 0.001	195.1 ± 73.2 *p* = 0.001

**Table 3:** Energy Metabolism and mitochondrial fuel oxidation.

Outcome Measures	Controls	PWH: 0 Weeks *p*-Value: HIV 0 w vs. Con	PWH: 12 Weeks *p*-Value: HIV 0 w vs. HIV 12 w	PWH: 20 Weeks *p*-Value: HIV 12 w vs. HIV 20 w
Fasted respiratory quotient (RQ)	0.76 ± 0.01	0.86 ± 0.04 *p* = 0.000	0.78 ± 0.03 *p* = 0.001	0.90 ± 0.05 *p =* 0.004
Fasted fatty acid oxidation (mg/kg lean mass/min)	1.39 ± 0.30	0.50 ± 0.20 *p =* 0.007	1.39 ± 0.09 *p =* 0.001	
Fasted carbohydrate oxidation (mg/kg lean mass/min)	0.80 ± 0.12	2.15 ± 0.34 *p =* 0.001	1.30 ± 0.45 *p =* 0.001	
Fasted fatty-acid oxidation (mg/kg/min)	0.96 ± 0.25	0.30 ± 0.11 *p =* 0.005	0.90 ± 0.15 *p =* 0.001	0.45 ± 0.23 *p =* 0.009
Fasted carbohydrate oxidation (mg/kg/min)	0.55 ± 0.11	1.32 ± 0.12 *p =* 0.001	0.84 ± 0.28 *p =* 0.001	1.24 ± 0.28 *p =* 0.002
Energy expenditure (kcal/d)	1637 ± 299	1538 ± 269 *p =* 0.4	1509 ± 232 *p =* 0.6	1592 ± 296 *p =* 0.2

**Table 4:** Tracer kinetic data and glycemic indices.

Outcome Measures	Controls	PWH: 0 Weeks *p*-Value: HIV 0 w vs. Con	PWH: 12 Weeks *p*-Value: HIV 0 w vs. HIV 12 w	PWH: 20 Weeks *p*-Value: HIV 12 w vs. HIV 20 w
**Tracer kinetic data:**				
Muscle protein breakdown rate (mg/kg LBM/h)	104.6 ± 23.7	145.4 ± 36.8 *p =* 0.003	96.3 ± 41.6 *p =* 0.027	
Glucose production rate (μmol/kg/min)	7.8 ± 1.4	7.7 ± 0.6 *p =* 0.83	7.5 ± 0.4 *p =* 0.4	
Rate of gluconeogenesis (μmol/kg/min)	5.6 ± 0.9	5.0 ± 0.8 *p =* 0.079	5.0 ± 0.4 *p =* 0.96	
Rate of glycogenolysis (μmol/kg/min)	2.2 ± 0.7	2.7 ± 0.5 *p =* 0.16	2.5 ± 0.3 *p =* 0.54	
**Glycemia and insulin resistance:**				
Fasting plasma glucose (mmol/L)	4.8 ± 0.4	5.5 ± 0.7 *p =* 0.048	4.8 ± 0.6 *p =* 0.000	5.5 ± 0.7 *p =* 0.002
Fasting plasma insulin (mU/L)	10.0 ± 2.7	56.8 ± 11.6 *p =* 0.000	20.4 ± 6.5 *p =* 0.000	26.3 ± 6.2 *p =* 0.039
Insulin resistance (HOMA-IR)	2.2 ± 0.7	13.7 ± 2.6 *p =* 0.000	4.3 ± 1.3 *p =* 0.000	6.3 ± 1.6 *p =* 0.002

**Table 5:** Inflammation, endothelial function, and genotoxicity.

Outcome Measures	Controls	PWH: 0 Weeks *p*-Value: HIV 0 w vs. Con	PWH: 12 Weeks *p*-Value: HIV 0 w vs. HIV 12 w	PWH: 20 Weeks *p*-Value: HIV 12 w vs. HIV 20 w
Plasma high-sensitivity IL-6 (pg/mL)	0.8 ± 0.2	4.9 ± 1.0 *p =* 0.000	1.7 ± 0.7 *p =* 0.000	2.5 ± 1.0 *p =* 0.003
Plasma TNFα (pg/mL)	33.8 ± 10.5	89.9 ± 15.1 *p =* 0.000	59.1 ± 8.0 *p =* 0.000	64.5 ± 7.4 *p =* 0.006
Plasma high-sensitivity C-Reactive Protein (ng/mL)	0.4 ± 0.0	2.2 ± 0.3 *p =* 0.000	1.1 ± 0.3 *p =* 0.000	1.3 ± 0.3 *p =* 0.034
Plasma sICAM1 (ng/mL)	364.9 ± 114.5	994.1 ± 222.8 *p =* 0.000	445.4 ± 99.9 *p =* 0.000	658.8 ± 104.8 *p =* 0.000
Plasma sVCAM1 (ng/mL)	627.1 ± 132.7	1643.9 ± 289.7 *p =* 0.000	976.5 ± 263.4 *p =* 0.000	1247.4 ± 245.1 *p =* 0.001
Plasma E-Selectin (ng/mL)	28.2 ± 7.6	62.8 ± 12.4 *p =* 0.000	41.1 ± 9.3 *p =* 0.000	52.1 ± 9.6 *p =* 0.000
Plasma 8-OHdG (pg/mL)	53.4 ± 4.8	182.0 ± 44.4 *p =* 0.000	54.9 ± 4.7 *p =* 0.000	115.3 ± 49.7 *p =* 0.01

**Table 6:** Physical function.

Outcome Measures	Controls	PWH: 0 Weeks *p*-Value: HIV 0 w vs. Con	PWH: 12 Weeks *p*-Value: HIV 0 w vs. HIV 12 w	PWH: 20 Weeks *p*-Value: HIV 12 w vs. HIV 20 w
Gait speed (m/s)	1.3 ± 0.1	1.0 ± 0.1 *p =* 0.005	1.3 ± 0.2 *p =* 0.003	1.1 ± 0.2 *p =* 0.016
Rapid 6-min walk (m)	644 ± 62	508 ± 23 *p =* 0.001	542 ± 23 *p =* 0.000	509 ± 37 *p =* 0.012
Chair-rise test (s)	18.9 ± 3.5	28.8 ± 3.8 *p =* 0.003	23.0 ± 3.0 *p =* 0.002	25.0 ± 2.6 *p =* 0.044
Muscle strength, dominant forearm (kg)	46.0 ± 13.0	31.3 ± 8.3 *p =* 0.004	39.0 ± 8.7 *p =* 0.023	33.7 ± 8.6 *p =* 0.028
Muscle strength, nondominant forearm (kg)	41.8 ± 12.5	27.3 ± 8.6 *p =* 0.009	35.3 ± 7.8 *p =* 0.022	30.4 ± 8.0 *p =* 0.061

**Table 7:** Cognition.

Outcome Measures	Controls	PWH: 0 Weeks *p*-Value: HIV 0 w vs. Con	PWH: 12 Weeks *p*-Value: HIV 0 w vs. HIV 12 w	PWH: 20 Weeks *p*-Value: HIV 12 w vs. HIV 20 w
Montreal cognitive assessment test (MoCA)	28.0 ± 1.6	21.1 ± 3.8 *p =* 0.002	25.0 ± 2.0 *p =* 0.005	26.0 ± 1.8 *p =* 0.17
Trails test A (sec)	34.6 ± 10.2	62.6 ± 17.4 *p =* 0.001	46.4 ± 12.6 *p =* 0.008	40.4 ± 11.2 *p =* 0.068
Trails test B (sec)	53.8 ± 20.4	117.5 ± 42.4 *p =* 0.003	69.8 ± 15.3 *p =* 0.007	86.0 ± 30.2 *p =* 0.13
Verbal-fluency test	41.0 ± 9.5	28.9 ± 9.2 *p =* 0.053	34.6 ± 5.6 *p =* 0.025	36.6 ± 5.5 *p =* 0.28
DSST: % completed	44.9 ± 7.7	31.3 ± 8.0 *p =* 0.012	37.1 ± 7.5 *p =* 0.015	36.1 ± 7.2 *p =* 0.47
DSST: % accuracy	99.0 ± 1.5	95.1 ± 4.2 *p =* 0.058	97.7 ± 3.2 *p =* 0.033	97.4 ± 4.3 *p =* 0.7
Plasma BDNF (ng/mL)	47.9 ± 9.6	26.3 ± 5.4 *p =* 0.003	47.2 ± 8.7 *p =* 0.000	36.7 ± 9.1 *p =* 0.005

**Table 8:** Body composition and blood pressure.

Outcome Measures	Controls	PWH: 0 Weeks *p*-Value: HIV 0 w vs. Con	PWH: 12 Weeks *p*-Value: HIV 0 w vs. HIV 12 w	PWH: 20 Weeks *p*-Value: HIV 12 w vs. HIV 20 w
Weight (kg)	91.5 ± 14.5	87.6 ± 8.6 *p =* 0.5	84.2 ± 9.7 *p =* 0.041	84.8 ± 11.6 *p =* 0.7
BMI	28.9 ± 2.7	29.5 ± 2.3 *p =* 0.7	28.3 ± 3.0 *p =* 0.04	28.3 ± 3.4 *p =* 0.6
Fat-mass (kg)	25.8 ± 6.7	29.9 ± 3.6 *p =* 0.2	27.6 ± 3.8 *p =* 0.047	
Truncal fat mass (kg)	12.7 ± 3.5	16.7 ± 2.7 *p =* 0.017	15.1 ± 2.7 *p =* 0.038	
Lean mass (kg)	63.1 ± 13.8	55.2 ± 9.9 *p =* 0.079	54.1 ± 10.8 *p =* 0.21	
Waist circumference (cm)	97.5 ± 6.7	105.4 ± 7.7 *p =* 0.10	98.1 ± 7.9 *p =* 0.000	100.3 ± 8.4 *p =* 0.031
Hip circumference (cm)	107.5 ± 8.6	106.4 ± 5.6 *p =* 0.81	106.4 ± 4.9 *p =* 0.96	106.4 ± 5.2 *p =* 0.99
Waist:Hip ratio	0.9 ± 0.0	1.0 ± 0.1 *p =* 0.016	0.9 ± 0.1 *p =* 0.000	0.9 ± 0.1 *p =* 0.053
Systolic BP (mm Hg)	120.8 ± 11.8	131.9 ± 8.8 *p =* 0.003	119.4 ± 14.3 *p =* 0.009	117.3 ± 14.8 *p =* 0.65
Diastolic BP (mm Hg)	75.6 ± 10.0	84.1 ± 8.5 *p =* 0.003	76.6 ± 9.9 *p =* 0.039	76.1 ± 9.6 *p =* 0.89

## Abbreviations

8OHdG	8-hydroxy-deoxyguanosine
BMI	Body mass index
CRP	C-reactive protein
DEXA	Dual-energy X-ray absorptiometry
DSST	Digital symbol-substitution test
FDG-PET	Fluorodeoxyglucose Positron Emission Tomography
GlyNAC	Combination of glycine plus N-acetylcysteine
GSH	Glutathione
GSSG	oxidized glutathione
HDL	High density lipoprotein
IL6	Interleukin 6
LDL	low density lipoprotein
LM	Lean mass
LC3A/B	Microtubule-associated protein 1A/1B-light chain 3
MFO	Mitochondrial fatty-acid oxidation
MGO	Mitochondrial glucose oxidation
MoCA	Montreal cognitive assessment
MPBR	Muscle protein breakdown rate
OxS	Oxidative stress
PGC1α	Peroxisome proliferator-activated receptor gamma coactivator 1-alpha
PINK	Phosphatase and tensin homolog (PTEN) induced kinase 1
PPARα	Peroxisome proliferator-activated receptor 1-alpha
PWH	Patients with HIV
RBC	Red-blood cell
ROS	Reactive-oxygen species
sICAM1	soluble intracellular adhesion molecule 1
sVCAM1	soluble vascular-cell adhesion molecule 1
SIRT3	Sirtuin 3
TBARS	thiobarbituric acid reducing substances
TNFα	Tumor-necrosis factor alpha
